# An Evidence Theory and Fuzzy Logic Combined Approach for the Prediction of Potential ARF-Regulated Genes in Quinoa

**DOI:** 10.3390/plants12010071

**Published:** 2022-12-23

**Authors:** Nesrine Sghaier, Jemaa Essemine, Rayda Ben Ayed, Mustapha Gorai, Riadh Ben Marzoug, Ahmed Rebai, Mingnan Qu

**Affiliations:** 1 National Nanfan Research Institute (Sanya), Chinese Academy of Agricultural Sciences, Sanya 572024, China; 2 CAS Center for Excellence in Molecular Plant Sciences, Institute of Plant Physiology and Ecology, Shanghai Institutes for Biological Sciences, Chinese Academy of Sciences, Shanghai 200032, China; 3 Laboratory of Advanced Technology and Intelligent Systems, National Engineering School of Sousse, Sousse 4023, Tunisia; 4 Department of Agronomy and Plant Biotechnology, National Institute of Agronomy of Tunisia (INAT), 43 Avenue Charles Nicolle, 1082 El Mahrajène, University of Carthage-Tunis, Tunis 1082, Tunisia; 5 Laboratory of Extremophile Plants, Centre of Biotechnology of Borj-Cédria, B.P. 901, Hammam Lif 2050, Tunisia; 6 Higher Institute of Applied Biology Medenine, University of Gabes, Medenine 4119, Tunisia; 7 Laboratory of Molecular and Cellular Screening Processes, Sfax Biotechnology Center, B.P 1177, Sfax 3018, Tunisia

**Keywords:** data fusion, machine learning, evidence theory, ARF-binding sites, *Chenopodium quinoa*

## Abstract

Quinoa constitutes among the tolerant plants to the challenging and harmful abiotic environmental factors. Quinoa was selected as among the model crops destined for bio-saline agriculture that could contribute to the staple food security for an ever-growing worldwide population under various climate change scenarios. The auxin response factors (ARFs) constitute the main contributors in the plant adaptation to severe environmental conditions. Thus, the determination of the ARF-binding sites represents the major step that could provide promising insights helping in plant breeding programs and improving agronomic traits. Hence, determining the ARF-binding sites is a challenging task, particularly in species with large genome sizes. In this report, we present a data fusion approach based on Dempster–Shafer evidence theory and fuzzy set theory to predict the ARF-binding sites. We then performed an “In-silico” identification of the ARF-binding sites in *Chenopodium quinoa*. The characterization of some known pathways implicated in the auxin signaling in other higher plants confirms our prediction reliability. Furthermore, several pathways with no or little available information about their functions were identified to play important roles in the adaptation of quinoa to environmental conditions. The predictive auxin response genes associated with the detected ARF-binding sites may certainly help to explore the biological roles of some unknown genes newly identified in quinoa.

## 1. Introduction

Natural systems, food security, and agricultural production have been adversely affected by devastating environmental changes [1]. Thus, the enrichment of our knowledge of plant systems will provide effective solutions and leading strategies for future plant yield improvement and breeding programs [2]. Particularly, quinoa constitutes among the tolerant plants to the challenging and harmful abiotic environmental factors [3]. Thanks to its nutritional characteristics and tolerance capacity to various environmental stress conditions, quinoa has become an attractive laboratory material for scientists and researchers worldwide. Hence, quinoa was selected as among the model crops destined for biosaline agriculture that could contribute to the staple food security for an ever-growing worldwide population under various climate change scenarios [4]. In these regards, the Food and Agricultural Organization of the United Nations (FAO) declared 2013 as the international quinoa year to shed light on this crop as it is an essential staple food rich in proteins and fibres which needs more attention to be paid and further research activities to be performed to well understand and better decipher its valuable potential in fighting starvation problems to ensure and consolidate a promising nutritional value for the human beings [5].

The severe environmental conditions and the climate changes variability accentuate the effects of numerous stresses on plants [1]. So far, to cope with the surrounding continuously changing environment, plants respond by significant rearrangements at the transcriptomic level and modulation in the expression level of a large number of stress-related genes. Thus, plant hormones have been reported as well to be involved in plant adaptation to different biotic and abiotic stress factors [6,7]. Notably, auxin plays a critical and pivotal role in improving plant tolerance by controlling the expression of many stresses’ responsive genes. Auxin signaling involves the activation or repression of gene expression by a specific class of ARF proteins that binds to the ARF-binding sites known as Auxin Response Elements, AuxREs [8,9]. The ARF-binding sites are the main contributors to the auxin response diversity. Thus, the knowledge and determination of the ARF-binding sites represent a key and significant step to understanding well and accurately determining the molecular basis of the auxin action, which could provide insights helping in plant breeding programs and thereby in the amelioration of certain agronomic traits. Therefore, inferring the presence of ARF-binding sites in the regulatory regions is essential both for functional and evolutionary analyses [9]. Thus, determining the ARF-binding sites constitutes a challenging task, particularly in species with large genome sizes.

Afterwards, a promising and powerful approach was used to determine the genome-wide ARF-binding site’s location. This approach has been extensively approved to be achieved through various experimental techniques, including ChIP-Chip [10,11], ChIP-Seq [12,13], and ChIP-Pet [14]. However, these experiments are time-consuming and require huge financial resources and support; additionally, their given results remain relative and depend on the conditions adopted and/or being used during the experimental procedure [15]. Nevertheless, a complementary and alternative approach mainly based on computational methods has recently emerged, which allows fast and efficient identification of the ARF-binding sites [16,17]. Hence, the computational prediction of the binding sites remains a pivotal goal in bioinformatics with great priority and interest.

Most of the computational methods have used the TGTC-containing consensus core sequences as a tool to detect the ARF-binding sites [9,18]. Furthermore, other employed methods are mainly based on the position weight matrices (PWMs) describing the sequence preference for the transcription factor [19]. Unfortunately, the predictions using merely the consensus motifs or PWMs often generate a large number of false positives. This makes the detection of the binding site spurious sometimes and inaccurate. Accordingly, some unavoidable challenges and substantial limitations should be considered and considered since there are often several false positive events that could be generated and/or detected. In addition, many variants of DNA-binding sequences exist and can be recognized by the ARF TFs. Therefore, a number of computational approaches have been proposed to overcome these limitations and constraints to improve the prediction of the ARF-binding sites [20].

In the same trend, several recent reports have used various machine learning approaches, including the support vector machine, SVM [21], random forest [22,23], deep learning [24], and convolutional neural network, CNN [25]. These methods employ mostly different lines of evidence for the prediction of the ARF-binding site, such as sequence conservation, gene ontology (GO), and location of the binding sites [20]. Some other methods have used the distance and number of the nearest histone modifications [26]. Other groups based on DNA use three-dimensional (3D) structural information to describe the binding specificities [27,28]. Nevertheless, certain models could predict the binding sites based on the ChIP-Seq data once available [26]. Many other methods relied on gene expression data to predict the binding sites [28,29,30].

With the increasing number of evidence sources for ARF-binding events, the adoption of computational methods for integrating these various data sources can further improve the prediction of TF binding [31]. Recently, substantial progress has been made and yielded new valuable insights concerning the ARF-binding activity. Thus, the ARF-binding site varies sequences, repeat numbers, spacing, and orientation, contributing to the binding specificity and affinities of the different ARF family members [32,33,34,35,36]. Hence, a combination of this genomic information with other data sets, such as gene expression, may improve the prediction of the ARF-binding events. Plants respond to different environmental stresses by regulating stress-responsive gene expression [37,38,39].

In this study, we present a data fusion approach-based DS evidence theory and fuzzy set theory [40]. Thus, we combined the predictive data extracted from two techniques frequently used in the detection of the binding sites. These two are the detection of overrepresented motifs and the linear discriminant analysis (LDA). From each method, we extracted several features and combined them with an orthogonal sum of the DST rule. The specific features of the ARF-binding sites are also integrated to further improve their prediction accuracy and reliability. Then, we evaluated our predictions using ChIP-Seq data from *Zea mays*. Subsequently, we performed an “In-silico” identification of the ARF-binding sites in Chenopodium quinoa. Quinoa (*Chenopodium quinoa* Willd.) is a halophytic pseudo-cereal crop that originated from the Andean region of South America [41]. It is an allotetraploid (2*n* = 4*x* = 36) with an estimated genome size of approximately 1.5 Gbp [42]. It is a more nutritious grain than any other major cereal [43,44,45].

## 2. Results

### 2.1. Modelling Approach

In this study, we perform a data fusion approach based on Dempster–Shafer theory and fuzzy set theory to predict ARF-binding sites. Thus, we combined different extracted features.

To meet our requirements, two hypotheses were considered herein, which are the following: “the motif can be an ARF-binding site” or “the motif can’t be an ARF-binding site”.

In terms of the DS evidence theory, we are located in the case where the frame of discernment is constructed with two single hypotheses, H1 and H2, then a single composite one (H3), unifying the two other hypotheses as mentioned in the following formula: H3 = H1 U H2 (union of H1 and H2), where H3 represents the ignorance indeed. The modelling of our method follows six major steps to represent the confidence in the detection of the ARF-binding site.

Step 1: Features determination.

Step 2: Construction of feature space.

Step 3: Determination of confidence regions.

Step 4: Modelling hypotheses.

Step 5: Fuzzification.

Step 6: Dempster–Shafer combination.

#### 2.1.1. Features Determination

The first step consists of extracting the different features that can be selected from each studied method. From the first method (detection of overrepresented motifs), we have extracted four features which are position (P), significance score (Sc), occurrence (O), and density (D). The position represents the initial feature that must be determined for each motif of the database that we enclosed. Thus, several regulatory elements have been identified in the 5 ′UTR regions [46,47]. In fact, we have chosen to use the position relative to the start codon ATG. However, the significance score (Sc) has been calculated using the Weeder algorithm as previously performed by [48]. The occurrence indicates the total number of the detected motifs (core sequences) in a whole genome. The density feature has been defined as the ARF-binding sites rate in the promoter of auxin responsive genes. In this context, we have selected the auxin response genes with twofold changes (FC) expression from the microarray data [49].

For the second method, an LDA has been performed using Z-curve features [50] and the GC% as potentially the discriminative features. Hence, the LDA represents among the most important supervised linear dimensional reduction techniques [51]. The Z-curve is a unique 3D representative curve of a DNA sequence (Equations (1)–(3)). Notably, the three Z-curve features used here are:

x1 = (a1 + g1) − (c1 + t1)(1)

y1 = (a1 + c1) − (g1 + t1)(2)

z1 = (a1 + t1) − (g1 + c1)(3)

#### 2.1.2. Construction of Feature Space

**Figure 1:**
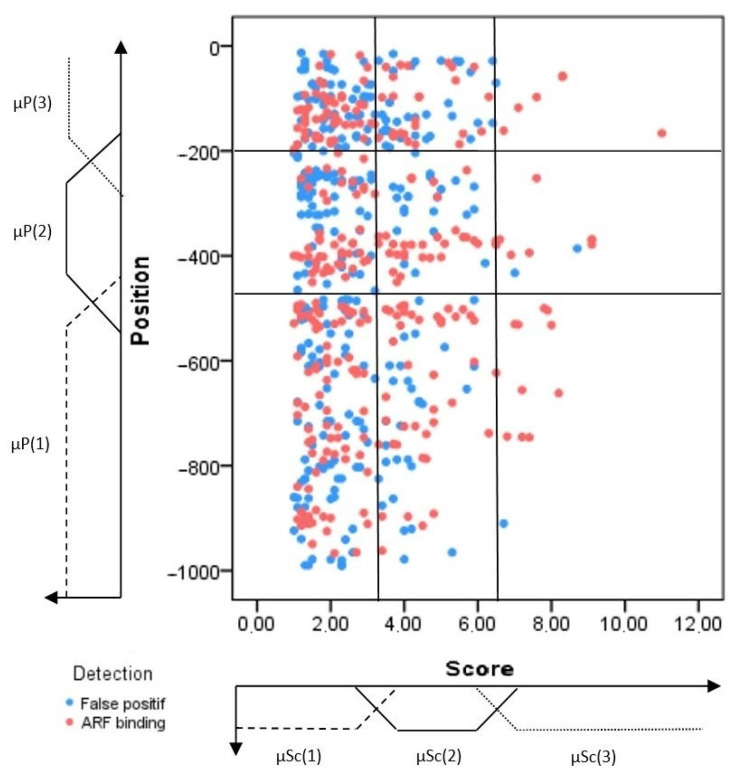
Feature space of the position and significance score representing the training sets distribution of the ARF-binding sites (red color) and false positives (blue color). Three fuzzy sets and their corresponding membership degree functions (μSc(i) and P(j)). The latter parameters were defined for each feature (position and score) and found to yield nine regions. The boundaries of the different sets were determined through a learning step as much as possible to define the discriminative regions.

**Figure 2:**
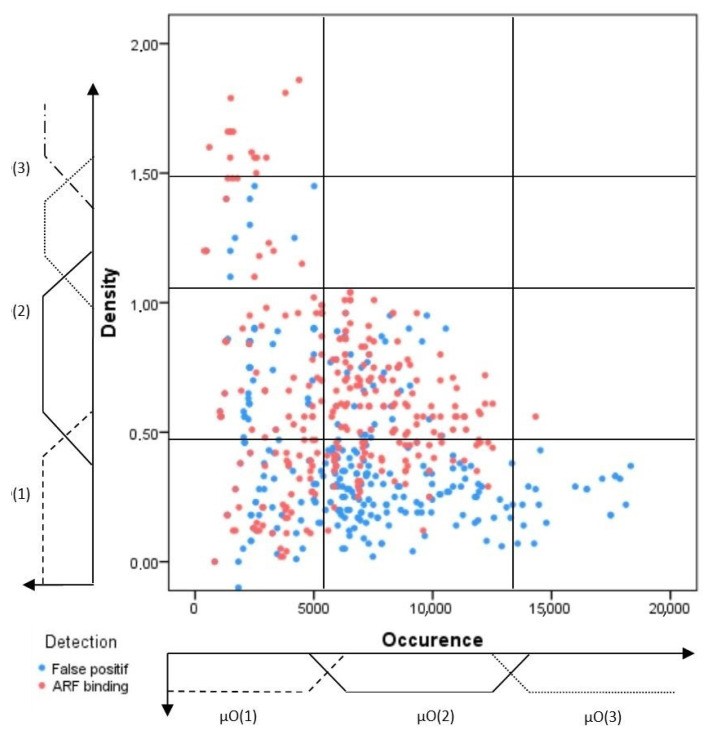
Feature space of the occurrence and density defining the training sets distribution of the ARF-binding sites (rx cq ed color) and false positives (blue color). Three fuzzy sets and their corresponding membership degree functions (μO(i) and D(j)). μO(i) and D(j) were defined for the occurrence and density, respectively, yielding twelve regions. The boundaries of the different sets were determined through a learning step as much as we could to find the discriminative regions.

**Figure 3:**
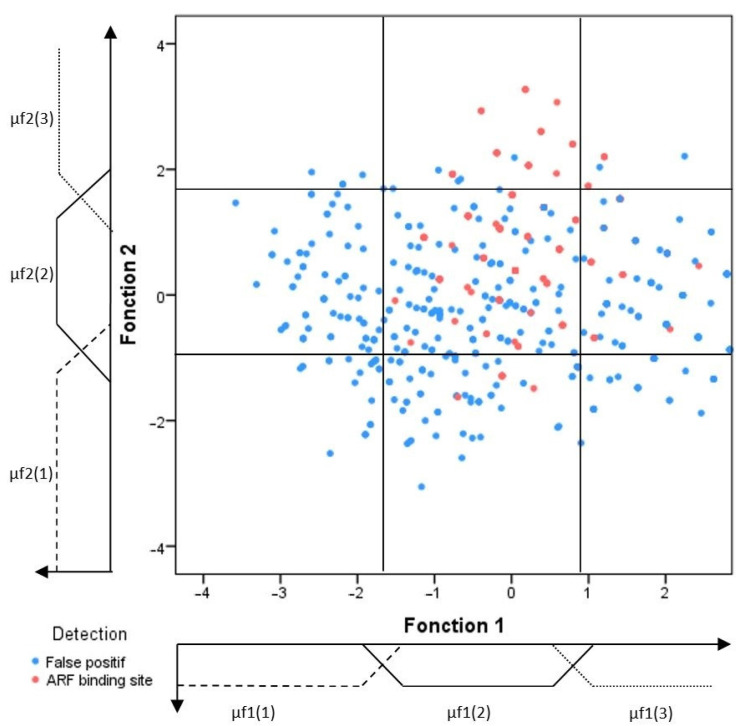
Feature space of the two first discriminative functions linear discriminant analysis of the represented training sets distribution of the ARF-binding sites (red color) and false positives (blue color). Three fuzzy sets and their corresponding membership degree functions (μF1(i) and F2(j)) were defined for each feature, yielding nine regions. The different sets of boundaries were determined through a learning step as much as possible to delimit discriminative regions.

In the following section, the training motifs have been divided into positive and negative training sets to construct a discriminative prediction model. These motifs are studied in the feature space, which helps to investigate the link between the selected features and the type of the considered motifs. Thereby, three learning graphs have been created (Figure 1, Figure 2 and Figure 3). These learning graphs represent the distribution of training motifs according to their calculated features. We have chosen to study independently the knowledge and insights acquired from, on one hand, the position and significance score (Figure 1) and, on the other hand, those provided by the Occurrence and Density (Figure 2) in order to distinguish as much as possible, the ARF-binding sites from the false positive’s events. For the LDA, we have used the Z-curve feature and the GC%. Function 1 and function 2 are the first and the second discriminant functions, respectively. The first function maximizes the differences between groups on that function. The second function maximizes differences in that function. So, the third learning graph depicts the distribution of the different types of motifs in function 1 versus function 2 feature space (Figure 3).

The first graph corresponds to the area of uncertainty that contains all types of regulatory elements. Thus, they are not discriminative features, as many ARF-binding sites were found in a very common region. Therefore, the interpretation of feature space relative to the two other features (occurrence and density) improves the TF classification and helps segregate or differentiate the ARF-binding sites, especially those found in the common region.

#### 2.1.3. Confidence Regions

Generally, the constructed learning graphs did not provide clear discrimination of the ARF-binding sites from the false positive hits. Indeed, for the sake of clarity, each graph was sub-divided into different areas called confidence regions that would be enriched by the ARF-binding sites. Each confidence region was defined according to the percentage of ARF-binding sites included. Mostly, this percentage varies from one region to another, and the graph partitioning was as illustrated in Figure 1, Figure 2 and Figure 3.

#### 2.1.4. Modelling Hypotheses

In order to perform automated detection processing for the ARF-binding sites, a confidence level should be automatically assigned for each unknown detected motif that can be placed on the graph. To achieve that, we have defined a gradual doubt through a set of four propositions:

- P1(Hi, Hj): Total ignorance
- P2(Hi, Hj): Low preference for the Hi hypothesis but high doubt between Hi and Hj
- P3(Hi, Hj): Strong preference for the Hi hypothesis but low doubt between Hi and Hj
- P4(Hi): Total confidence in the Hi hypothesis, no doubt

Table 1 shows the seven cases representing the various preference degrees that enable expressing the hesitation between the two hypotheses (H1 and H2).

Thereafter, all propositions are interpreted in a numerical form for which the information sources can provide evidence by using elementary mass values. The preference levels from P1 to P4 are modeled by a gradual mass value and are equal to 0, 0.33, 0.67, and 1, respectively [52]. P4 represents the hypothesis with total confidence and no doubt that the detected motif is an ARF-binding site. In the absence of doubt, the mass value assigned is equal to 1. The mass value corresponds to the total doubt equals zero. The transformation of doubt into a quantitative mass value was provided in detail in Table 1. Afterwards, a proposition was assigned to each confidence region from the previous analysis of the learning graphs based on the percentages of ARF-binding sites already existing in each region.

#### 2.1.5. Fuzzification

In the previous sections, we applied a discrete representation to define the regions of the learning graphs. This representation assigns different classifications for the close motifs from both the boundaries sides. However, the boundaries between regions were not well defined, and the transition from one region to another remains tricky and ambiguous. Thus, to further describe this fuzziness aspect in the learning graphs when using the mass functions, we suggest incorporating the theory of fuzzy logic. Accordingly, a gradual, continuous, and smooth transition between regions can be achieved using the membership function concept. Therefore, we have defined the fuzzy sets for each measured feature. For instance, for the feature significance score (Sc), four distinct sets (small, average, high, and very high) were defined and recognized. Thus, a smooth transition from one region to another can merely occur. During the learning stage, such a detected motif could be weighted by its membership degrees to different fuzzy sets and characterized by a mass value according to the doubt level for the hypothesis related to its corresponding region.

For each detected motif, three masses were calculated according to Equations (4)–(6) below, corresponding to the three learning graphs (Figure 1, Figure 2 and Figure 3). They are given, respectively, as follows:

(4) $$m\left(O\in S/Sc\&P\right)=\sum_{i=1,j=1}^{i=3,j=3}\mu_{Sc\left(i\right)}\left(x\right)\times \mu_{p\left(j\right)}\left(y\right)\times m_{R_{ij}}\left(O\in S/Sc\&P\right)$$

(5) $$m\left(O\in S/O\&D\right)=\sum_{i=1,j=1}^{i=3,j=4}\mu_{O\left(i\right)}\left(x\right)\times \mu_{D\left(j\right)}\left(y\right)\times m_{R_{ij}}\left(O\in S/O\&D\right)$$

(6) $$m\left(O\in S/f1\&f2\right)=\sum_{i=1,j=1}^{i=3,j=3}\mu_{f1\left(i\right)}\left(x\right)\times \mu_{f2\left(j\right)}\left(y\right)\times m_{R_{ij}}\left(O\in S/f1\&f2\right)$$

where *S* represents any sub-set of the hypotheses, $m_{R_{ij}}\left(O\in S/Sc\&P\right)$, $m_{R_{ij}}\left(O\in S/O\&D\right)\;$, and $m_{R_{ij}}\left(O\in S/f1\&f2\right)\;$ designates the mass corresponding to the region *Rij* of the significance score/position graph, Occurrence/Density graph, and *f*1/*f*2 graph, respectively.

#### 2.1.6. Dempster–Shafer Combination

Subsequently, the data fusion step consists of a combination of the confidence levels deduced from the two methods of detection. Firstly, we must combine the two masses of method 1 (Equations (4) and (5)). Thus, the mass function was obtained by fusing the two masses from the two learning graphs of method 1 by using the orthogonal sum of DS evidence:

(7) $$m_{1}\left(O\in S\right)=m\left(O\in S/Sc\&P\right)⊕m\left(O\in S/O\&D\right)$$

Then, the final mass function (*m_fusion_*) (Equation (8)) was defined by fusing the two masses $m_{1}\left(O\in S\right)$ (Equation (7)) and m(O∈S/f1&f2) (Equation (6)) as shown in the following equation:

(8) $$m_{fusion}\left(O\in S\right)=m_{1}\left(O\in S\right)⊕m\left(O\in S/f1\&f2\right)$$

Thus, this approach integrates many specific features of the ARF-binding sites, such as sequence conservation, cut counts in a 200 bp window around the site, location relative to the transcription starting sites, and motif orientation and spacing. The number of motifs repeats and their spacing represents a very characteristic and major feature for the ARF-binding sites. In this regard, Galli and co-workers have demonstrated that the ARFs bind more frequently with high affinity to the sites containing multiple TGTC core sequences in *Zea mays* [32]. The same authors found that most of the peaks containing two or more TGTCs (55–86%) and/or the peaks with higher TGTC motif numbers showed stronger peak signal intensity [32]. Conversely, the randomly selected genomic regions contain a much lower percentage of fragments with two or more TGTC motifs and a much higher proportion of instances with zero or only a single TGTC core sequence [32]. Additionally, motif orientation and spacing are both important features in the ARF-specific binding sites. Overall, 90% of total peaks reveal less than 50 intervening nucleotides for all orientations, while 29~46% showed spacing of fewer than 20 nucleotides [32,34]. These findings are like previous studies performed on *Arabidopsis thaliana* [53,54]. The integration of these important features reduces the prediction space and the potential of false positive rates, thereby promoting prediction reliability and precision.

### 2.2. Evaluation of Data Fusion Approach on the Experimental Data

In order to assess the predictive accuracy of our methodology, we constructed gold-standard datasets for six ARFs which are ARF4, ARF13, ARF14, ARF18, ARF35, and ARF39. The ChIP-Seq data from *Zea mays* was used to evaluate and/or test the performance of the data fusion method for identifying the ARF-binding sites. These ARFs members were chosen because they are available in a narrow peak format with peak summit values. Therefore, we used the middle 100 bases of each peak to ensure including enough sequence length in identifying the ARF-binding sites while minimizing the false detections. All ChIP-Seq peaks of the ARFs are taken (considered) as positive binding events. The positive sequences correspond to 50 bases from each side of the maximum signal for each ChIP-Seq peak. The control set contains randomly generated, non-overlapping peaks harboring the same mean peak width as the positive ones (peaks).

**Figure 4:**
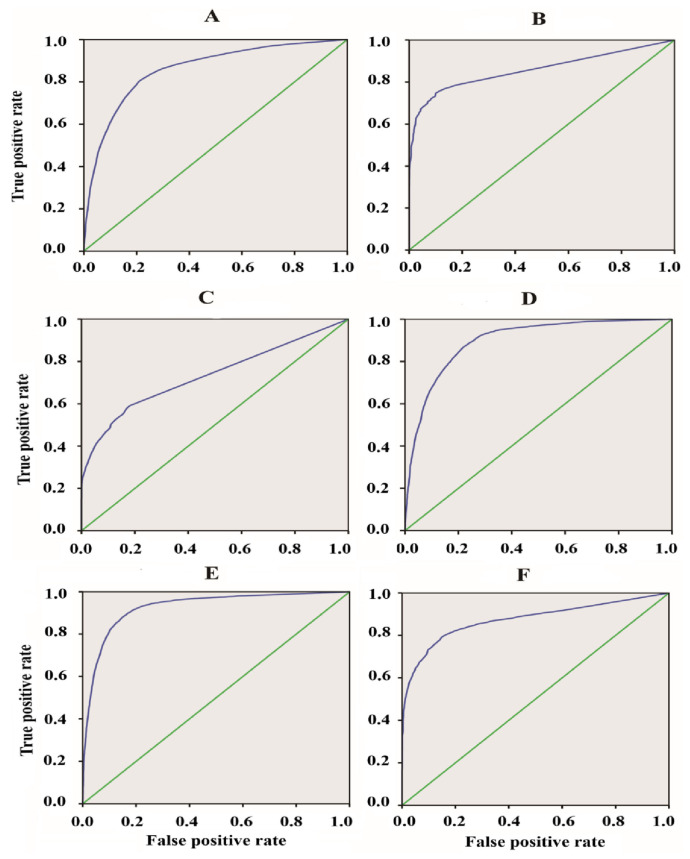
The receiver operating characteristic (ROC) curves of ARF-binding sites predicted using our data fusion method. The ARF members (4, 13, 14, 18, 35, and 39) were depicted by the alphabetic letters from (**A**–**F**), respectively. The true positive rate was evaluated as follows: TPR = TP/(TP + FN) and the false positive one FPR = TN/(TN + FP). The reference lines are displayed in green color for all the ARFs members.

The receiver operation characteristic curve (ROC) can be performed by plotting the true positive rate against the false positive one (rate) at different thresholds. Thus, we mainly considered the area under the ROC curve (AUC) to assess the aggregated classification performance. Figure 4 and Table 2 display the ROC curves and AUC values, respectively, for the six evaluated ARFs members. The data fusion-based algorithm could discriminate the ChIP-Seq peaks from the control sequences for the whole evaluated ARFs to some extent (degree), as evidenced by the fact that the AUC scores of all the ARFs members surpassed the random expectation of 0.5.

**Figure 5:**
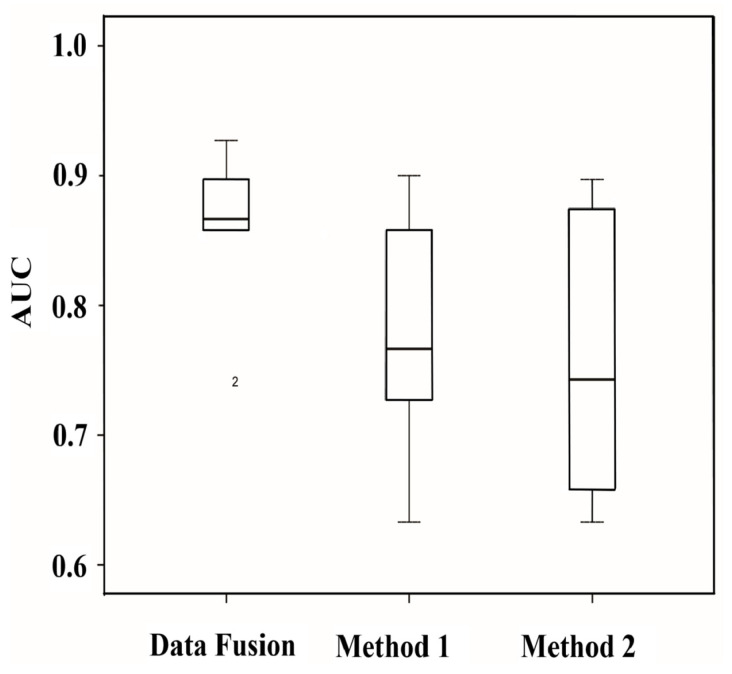
Boxplot of the average AUC of 6 different ARFs members studied herein before and after combination using specific features of ARF-binding. Data fusion method is a combination of method_1 and method_2. Method_1 represents the prediction using overrepresented motifs, and method_2 corresponds to the prediction based on linear discriminant analysis (LDA).

In order to investigate the influence of the combination by data fusion approach, we presented in the boxplots of Figure 5 the average AUC for the 6 studied ARFs members before and after combination using specific features such as the number of motifs repeats and their spacing. Notably, the comparison of the AUC values obviously shows that the data fusion method greatly outperforms the other prediction methods (Figure 5). This reveals the utility of our proposed methodology for the recognition of the ARF-binding sites. Hence, our results depicted in Figure 5 clearly show that using the combination of specific features substantially reduced the number of false positives. Thus, the average AUC of our used method ranged from 0.85 to 0.93, and this reflects the high efficiency of this method and the low rate of spurious combination events generated during our implemented method.

### 2.3. Comparison of Our Approach to Other Methods

**Figure 6:**
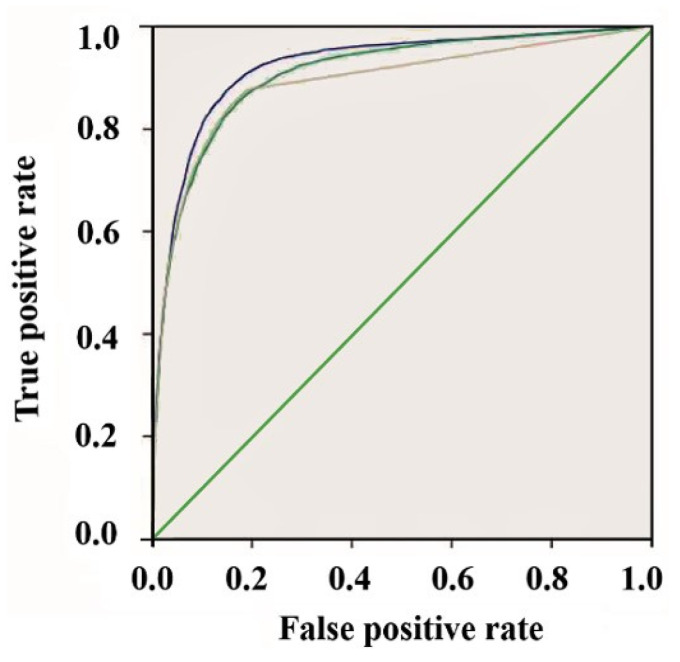
The accuracy of Data fusion methodology (blue curve) was compared to Fimo (green curve) and Matrix scan (yellow curve) using ROC curves for the ARF39 binding sites. Higher curve (close to the top left corner) represents the ROC curve of our method corresponding to the model with better ARF-binding sites prediction quality.

In order to evaluate the performance of the data fusion approach for identifying the ARF-binding sites, we compared our methodology with other TFs site prediction software, such as Fimo, as well as Matrix scan. For example, we obtained ChIP-Seq data for the ARF39 and compared the performances and reliabilities of the programs to detect the true positive ARF-binding sites using ROC curves (Figure 6). The area under the curve (AUC) was calculated for each program as well as area under precision recall curve (AUPR). The AUPR evaluates the classification performance in terms of precision and recall.

In comparison to the evaluated tools using ARF39 ChIP-Seq data, our method, represented by the higher curve close to the top left corner, shows the best fit (high efficiency) to predict with high accuracy the true positive ARF sites (Figure 6; blue color curve). Our model also achieves higher overall AUC than all the other previously tested methods (Fimo, Matrix scan). As well, the AUPR comparison (Table 2) clearly indicates that our model performs well in terms of the area under the precision-recall curve. This reveals that our model exhibits better performance for the AUPR if compared to Fimo and Matrix scan.

To evaluate our model, we have compared our prediction to previously published detected auxin binding sites in quinoa. A valuable recent study by Yu et al. identified four auxin-responsive elements in the promoter of AUR62002523, AUR62002810, AUR62004953, and AUR62004956 genes. Our analysis also identified with a high score these AuxRE as ARF-binding sites. The high scores are explained by the fact that the studied genes by Yu et al. are Auxin Response Protein (AUX/IAA) since our model takes into consideration the genes’ expression levels in response to auxin.

Another recent study by Zhu et al. identified several cis-acting elements associated with auxin in the promoter region of the quinoa SRS genes. The authors highlight the importance of Cis-acting elements in plant defense against various biotic and abiotic stresses. Auxin response elements have been particularly detected in the promoters of three CqSRS gene family members, which are CqSRS 4 (AUR62007636), CqSRS 5 (AUR62007664), and CqSRS 8 (AUR62016794). These AuxRe are likewise identified as ARF-binding sites by our model. All studied cis elements are detected par data fusion model. In addition, some other motifs are also identified as ARF-binding sites. As an example, we detect a second reverse motif at the −354 bp position of the AUR62004956 gene. The spacing between the detected motifs is about thirty bp [55,56].

### 2.4. Functional Annotation of ARF-Binding Sites in Chenopodium quinoa

**Figure 7:**
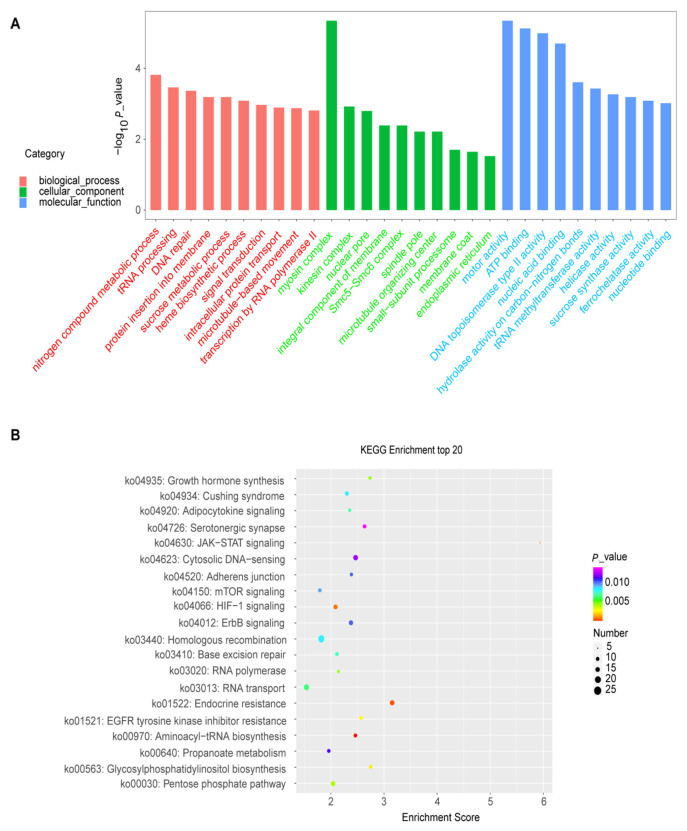
GO and KEGG analyses performed on the ARF4. (**A**), GO analysis showing the top 20 enriched pathways for the biological process, cellular component, and molecular function. (**B**), KEGG analysis displaying the top 20 enriched metabolic pathways based on the ARF4 gate.

**Figure 8:**
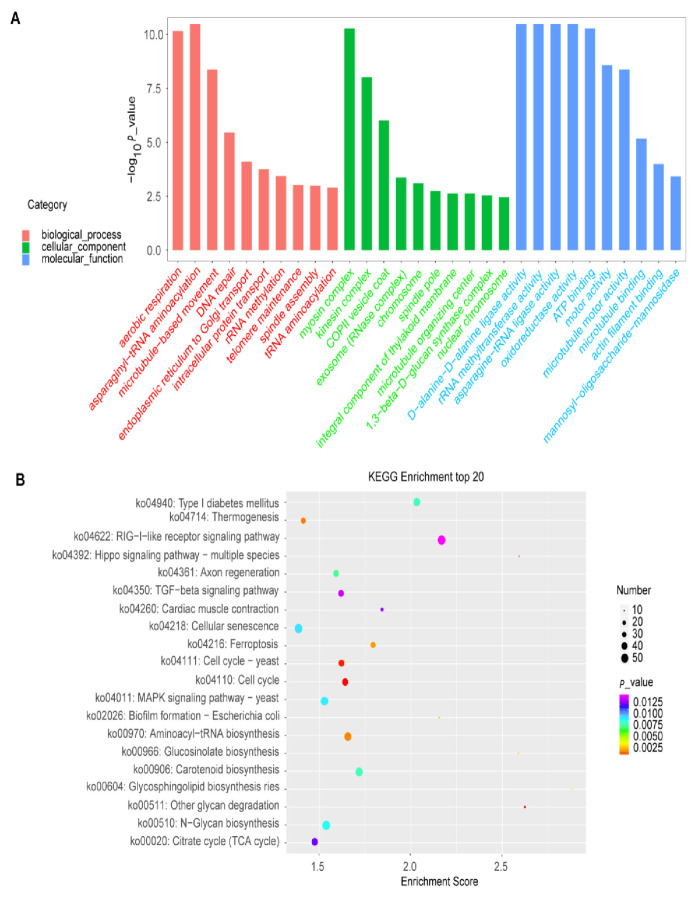
GO and KEGG analyses performed on the ARF39. (**A**), GO analysis displaying the top 20 enriched pathways for the biological process, cellular component, and molecular function. (**B**), KEGG analysis displaying the top 20 enriched metabolic pathways based on the ARF39 gate.

To further explore the gene function and gain more insights into the biological pathways implicated in the auxin response in quinoa, we conducted the GO enrichment and KEGG analysis to illustrate the auxin-responsive genes harboring potential ARF-binding sites in their promoters’ regions (Table S1). To unravel the major auxin response processes, three top biological pathways from the enriched analysis were selected for further investigation. In terms of GO enrichment, we found that the proteins showing significantly enriched expression were involved in three top pathways of the biological process of ARF4 (nitrogen compound metabolic process, tRNA processing, and DNA repair; Figure 7A; Table S2), ARF13 (IMP salvage, (1→3)-beta-D-glucan biosynthetic process and endoplasmic reticulum to Golgi vesicle-mediated transport; Figure S1A; Table S2) and ARF14 (telomere maintenance, DNA repair, and rRNA methylation; Figure S2A; Table S2), ARF18 (regulation of DNA replication, DNA damage checkpoint, and chromosome organization; Figure S3A; Table S2), ARF35 (regulation of DNA replication, DNA damage checkpoint, and chromosome organization; Figure S4A; Table S2) and ARF39 (aerobic respiration, asparaginyl-tRNA amino-acylation, and microtubule-based movement; Figure 8A; Table S2). For the other pathways, complete and detailed descriptions are given in Table S2.

The KEGG analysis reveals that various metabolic pathways were enriched regarding the up-regulated genes by elevated CO_2_. These pathways include starch and sucrose metabolism, Fatty acid biosynthesis, Nitrogen metabolism, Seleno-compound metabolism, Amino-acyl-tRNA biosynthesis, and Carotenoid biosynthesis (Figure 7B, Figure 8B and Figures S1B–S4B; Table S3). For the other pathways, complete and detailed descriptions are given in Table S3.

## 3. Discussion

In this study, we developed a new algorithm for the prediction of ARF-binding sites by combining a set of genomic features extracted from two basic methods (overrepresented motifs and LDA). To achieve that, we attempted to extract the features from the existing ARFs ChIP-Seq data based on the two chosen methods, and we built up the model by applying a data fusion approach. The supervised learning model step constitutes an important and pivotal task of the work. We then extensively evaluated the transferability using this algorithm and found that the learned model was well accomplished in predicting the ARF-binding sites with high accuracy and reliability.

Quinoa (*Chenopodium quinoa* Willd.) is a pseudo cereal of the Amaranthaceae family, which originates from the Andean region and can adapt to different edaphic and climatic conditions. Both quinoa seeds and leaves are edible parts of the plant; however, the seeds are considered most in terms of economic and scientific aspects. It is a seed crop with high nutritional value since seeds are rich in proteins, lipids, fiber, vitamins, and minerals and have a remarkable balance of essential amino acids. Moreover, due to the absence of gluten, quinoa constitutes a suitable diet for celiac patients or gluten-related disorders [57]. Thus, the nutritional value of quinoa seeds has been reported to meet, and even exceed, that recommended by the World Health Organization, WHO [58]. Furthermore, the quinoa plant is resistant to various environmental stresses, including cold [59], salt [60], and drought [61]. Very likely, for these reasons, quinoa has been called since a while the “golden grain” [57].

In the same trend, the GO enrichment reveals that the various pathways were enriched regarding the list of genes containing the predictive ARF-binding sites in their promoters. Some pathways are known to be involved in the auxin signaling and response in other plants, such as Arabidopsis [62], rice [63], and tea [64]. These results corroborate our prediction reliability based on the data fusion approach and potentially suggest the implication of these pathways in auxin response in *Chenopodium quinoa* as well. For instance, the myosin complex pathway was highly enriched in the following ARFs: ARF4, ARF13, and ARF39 (Figure 7, Figure 8 and Figure S1A). In line with our findings, a recent work also suggested that the myosin XIs could play a significant role in the auxin regulation network in *Arabidopsis thaliana* [62]. Hence, the myosin XIs were found to be involved in mediating and orchestrating (in a concerting manner) the root organogenesis via their effects on the polar distribution of auxin responses and on the cell division process [62].

Besides, another interesting significantly enriched pathway was related to the tRNA amino-acylation and processing. In line with our findings, recently, Chen and co-workers have found that the tRNA modification plays an essential role in auxin signaling in rice plants in response to a moderately high temperature of about 35 °C [63]. Our results confirm and agree with this funding and suggest the implication of tRNA processing in the auxin signalling output in quinoa under heat-stress conditions. Furthermore, the GO enrichment exhibits that the nitrogen compound metabolic process was enriched regarding the genes containing ARF4 binding sites in their promoters (Figure 7A). In the same trend, a recently published study reported that the lateral root formation could be induced by a low nitrogen (N)concentration via auxin biosynthesis and accumulation in tea plants [64]. In this regard, our results strongly also suggested the implication of the ARF4 family in this process (lateral root formation under low N) in quinoa.

Some other pathways were highly enriched in the predicted auxin-responsive genes but have no or less information about their functional annotations and relation to auxin response. Thus, our results proposed that these pathways play crucial or prominent roles in the auxin signaling and adaptation of quinoa to changing environmental conditions. Among these pathways, the kinesin complex was significantly enriched in the list of all the studied ARFs response genes (ARF4, ARF13, ARF14, ARF18, ARF35, and ARF39) (Figure 7A, Figure 8A and Figures S1A–S4A). This suggests a potentially prominent role of kinesin in the auxin signaling process. The (1→3)-beta-D-glucan biosynthetic process was also enriched in the ARF13 gene list. This (1→3)-beta-D-glucan is highly represented in monocotyledons and known to be involved in plants’ response to oxidative and heat stresses [65,66,67]. Our analyses suggest thus the involvement of this pathway in quinoa adaptation to oxidative and heat stresses by implying the ARF13 auxin signaling (Figure S1A). Hence, various pathways were enriched regarding the list of genes containing the predictive ARF-binding sites in their promoters, including telomere maintenance, DNA repair, asparaginyl-tRNA amino-acylation, and chloroplast movements (Table S2). These different pathways were extensively reported to be implicated in the *Chenopodium quinoa* tolerance to adverse environmental factors, such as salinity [3,68,69], drought [70], or their combined effect [71,72]. Eventually, this funding may help scientists to better understand and identify the crucial molecular mechanisms of the auxin action and to further uncover how the auxin signaling pathway could involve, in the quinoa plant, against different abiotic stresses such as salinity since it (quinoa) has earlier been widely used as a model crop for understanding the salt-tolerance in halophytes [4]. In addition, the predictive auxin response genes with the detected ARF-binding sites could help explore the molecular functions and the biological roles of some unknown genes in quinoa since its genome has still not yet sequenced till date, which makes it tough and delicate to decipher its deep genomic functions with precision. One of the main advantages of Dempster–Shafer theory (DST) is that we can utilize it to generate a degree of belief by taking all the evidence into account. This evidence can be obtained from different sources. Merge several types of information specific to ARF-binding sites to reduce false positives significantly. However, it still needs to be improved by introducing other parameters like digital genomic footprinting or DNase-I hypersensitivity score [73]. Further directions will be the development of algorithms by fusion more specific and recently investigated to predict ARF-binding sites.

## 4. Material and Methods

### 4.1. Training Set

A set of validated ARF-binding sites was collected from the published data online and ChIP-Seq data for six of the *Zea mays* ARF-binding sites regions from the gene expression omnibus (GEO) under the accession number GSE111857 [32,34].

The data were available in narrow peak format with peak max values. We extracted the binding peaks for each dataset with a length of 100 bp centering on the summit of the originally called binding peaks. We divided our dataset into training and test sets. The whole genomes dataset and upstream sequences of *Zea mays* and *Chenopodium quinoa* were downloaded from phytozome (www.phytozome.org (accessed on 18 September 2019)) and used for all our analysis below.

A Linear discriminant analysis (LDA) was performed using SPSS (v. 16.0, statistical package for the social sciences, Chicago, IL, USA). The microarray data of the primary response to auxin in Arabidopsis was taken from the Genevestigator database (https://genevestigator.com/gv/ (accessed on 18 November 2019)) [74].

### 4.2. Algorithm Implementation

The main algorithm was implemented under the R software environment language version R-3.5.3. All computations were performed on a single CPU Intel Core i7 computer running at 2.8 GHz, with 8 GB main memory. The source code is available upon request.

In order to assess the predictive accuracy of our methodology, we constructed gold-standard datasets for six ARFs which are ARF4, ARF13, ARF14, ARF18, ARF35, and ARF39.

### 4.3. Evaluation of Data Fusion Approach

The ChIP-Seq data from *Zea mays* was used to evaluate and test the performance of the data fusion method for identifying the ARF-binding sites. The gold-standard data set was constructed based on ChIP-Seq data of the given ARFs currently under investigation. These ARFs members were chosen because they are available in a narrow peak format with peak summit values. Therefore, we used the middle 100 bases of each peak to ensure including enough sequence length in identifying the ARF-binding sites while minimizing the false detections. All ChIP-Seq peaks of the ARFs are considered positive binding events. The positive sequences correspond to 50 bases from each side of the maximum signal for each ChIP-Seq peak. The control set contains randomly generated, non-overlapping peaks harboring the same mean peak width as the positive ones.

Thus, we mainly considered the area under the ROC curve (AUC) to estimate the aggregated classification performance. The receiver operation characteristic curve (ROC) and average Area Under the Precision-Recall curve (AUPR) can be performed by plotting the true positive rate against the false positive one at different thresholds.

### 4.4. Functional Annotation

To further explore the gene function and gain more insights about the biological pathways implicated in the auxin response in quinoa, we conducted the GO enrichment and KEGG analysis to illustrate the auxin-responsive genes containing potential ARF-binding sites in their promoters’ regions.

A multi-omics data analysis tool, OmicsBean (http://www.omicsbean.cn (accesssed on 1 July 2020)), dedicated to integrating the gene ontology (GO) enrichment and Kyoto encyclopedia of genes and genomes (KEGG) pathway analysis, was employed to investigate the obtained gens lists (Table S2). A *p*-value < 0.05 (Fisher’s exact test) was considered as the threshold to determine the significant enrichments for the GO and KEGG pathways.

## 5. Conclusions

In this article, we present a new data fusion approach based on DS evidence theory and fuzzy set theory to predict the ARF-binding sites in quinoa. To achieve that, we developed a new algorithm for the prediction of ARF-binding sites by combining a set of genomic features extracted from two basic methods (overrepresented motifs and LDA). Thus, we found that the learned model was well accomplished in predicting with high accuracy and reliability these ARFs. Afterwards, we performed an “In-silico” identification of the ARF-binding sites in *Chenopodium quinoa*. The GO enrichment corroborates that various biological pathways were enriched regarding the list of genes containing the predictive ARF-binding sites in their promoters. These pathways were identified to play important roles in the auxin signaling and adaptation of quinoa to severe environmental conditions.

## Figures and Tables

**Table 1:** Seven possibilities for the various preference degrees expressing the hesitation between the two hypotheses, H1 and H2.

Proposition	m(H1) (AuxRE)	m(H2) (Pas AuxRE)	m(H1 U H2) (Ignorance)
P1(H1, H2)	0	0	1
P2(H1, H2)	0.33	0	0.67
P3(H1, H2)	0.67	0	0.33
P4(H1)	1	0	0
P2(H2, H1)	0	0.33	0.67
P3(H2, H1)	0	0.67	0.33
P4(H2)	0	1	0

**Table 2:** Area under the receiver operator characteristic (ROC) curve (AUC) and average Area Under the Precision-Recall curve (AUPR) for six evaluated ARFs: ARF4, ARF14, ARF35, ARF39, ARF13, and ARF18.

ARF	ARF4	ARF14	ARF35	ARF39	ARF13	ARF18
**AUC**	0.859	0.733	0.927	0.874	0.858	0.897
	**Data fusion**		**Matrix scan**		**Fimo**	
**AUPR**	0.91		0.8		0.78	

## Data Availability

The data used to support the findings of this study are included within the article.

## Supplementary Materials

The following supporting information can be downloaded at: https://www.mdpi.com/article/10.3390/plants12010071/s1, Figure S1: GO and KEGG analyses performed on the ARF13. A, GO analysis displaying the top 20 enriched pathways for the biological process, cellular component and the molecular function. B, KEGG analysis displaying the top 20 enriched metabolic pathways based on the ARF13 gate; Figure S2: GO and KEGG analyses performed on the ARF14. A, GO analysis displaying the top 20 enriched pathways for the biological process, cellular component and the molecular function. B, KEGG analysis displaying the top 20 enriched metabolic pathways based on the ARF14 gate; Figure S3: GO and KEGG analyses performed on the ARF18. A, GO analysis displaying the top 20 enriched pathways for the biological process, cellular component and the molecular function. B, KEGG analysis displaying the top 20 enriched metabolic pathways based on the ARF18 gate; Figure S4: GO and KEGG analyses performed on the ARF35. A, GO analysis displaying the top 20 enriched pathways for the biological process, cellular component and the molecular function. B, KEGG analysis displaying the top 20 enriched metabolic pathways based on the ARF35 gate; Table S1: List of putative auxin response genes containing ARF-binding sites in their promoters regions predicted by data fusion approach; Table S2: Enriced biological pathways based on gene ontology (GO) analysis in *Chenopodium quinoa*; Table S3: Enriced biological pathways based on Kyoto encyclopedia of genes and genomes (KEGG) analysis in *Chenopodium quinoa*.

## Abbreviations

ARF auxin-responsive transcription factor; AUC, area under receiver operation characteristic curve; AUPR, area under precision-recall curve; AuxRE, auxin response element; ChIP-Chip, chromatin immuno-precipitation followed by microarray hybridization; ChIP-PET, chromatin immuno-precipitation with the paired-end tags; ChIP-Seq, chromatin immuno-precipitation sequencing; CNN, convolutional neural network; DNA, deoxyibo-nucleic-acid; DST, Dempster–Shafer theory; FAO, food and agricultural organization; GEO, gene expression omnibus; GO, gene ontology; KEGG, Kyoto encyclopedia of genes and genomes; LDA, linear discriminant analysis; PWM, position weight matrices; ROC, receiver operating characteristic; SVM, support vector machine; TF, transcription factor.
