# Supplementary material for: An Evidence Theory and Fuzzy Logic Combined Approach for the Prediction of Potential ARF-Regulated Genes in Quinoa
Source: Plants (Basel). 2022 Dec 23;12(1):71. doi: 10.3390/plants12010071 (PMC9824623; doi:10.3390/plants12010071)
Supplement: Supplementary file 1 [file plants-12-00071-s001.zip › plants-2068184-Supplementary Figures.pdf]

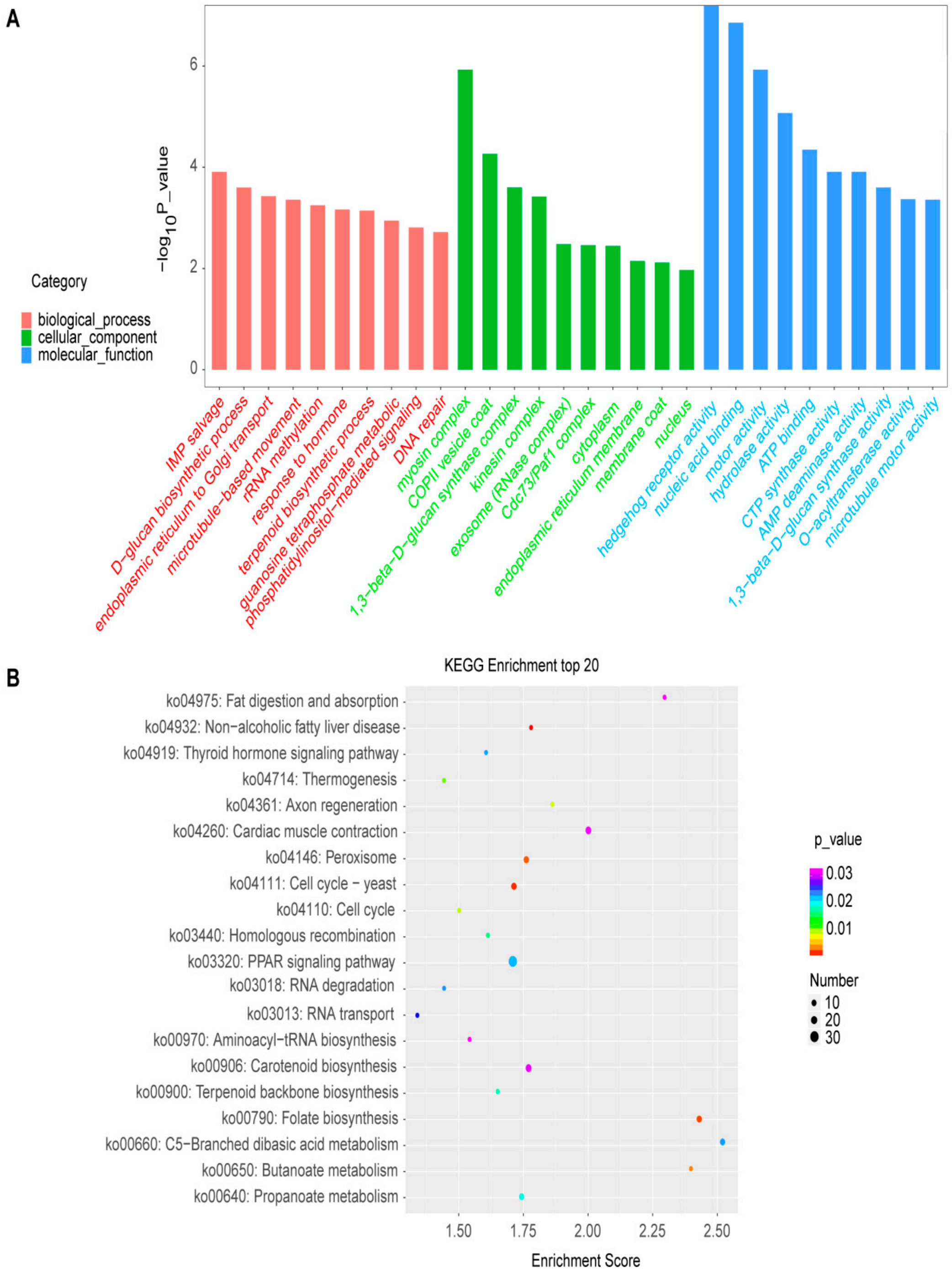

Figure S1: GO and KEGG analyses performed on the ARF13. A, GO analysis displaying the top 20 enriched pathways for the biological process, cellular component and the molecular function. B, KEGG analysis displaying the top 20 enriched metabolic pathways based on the ARF13 gate.

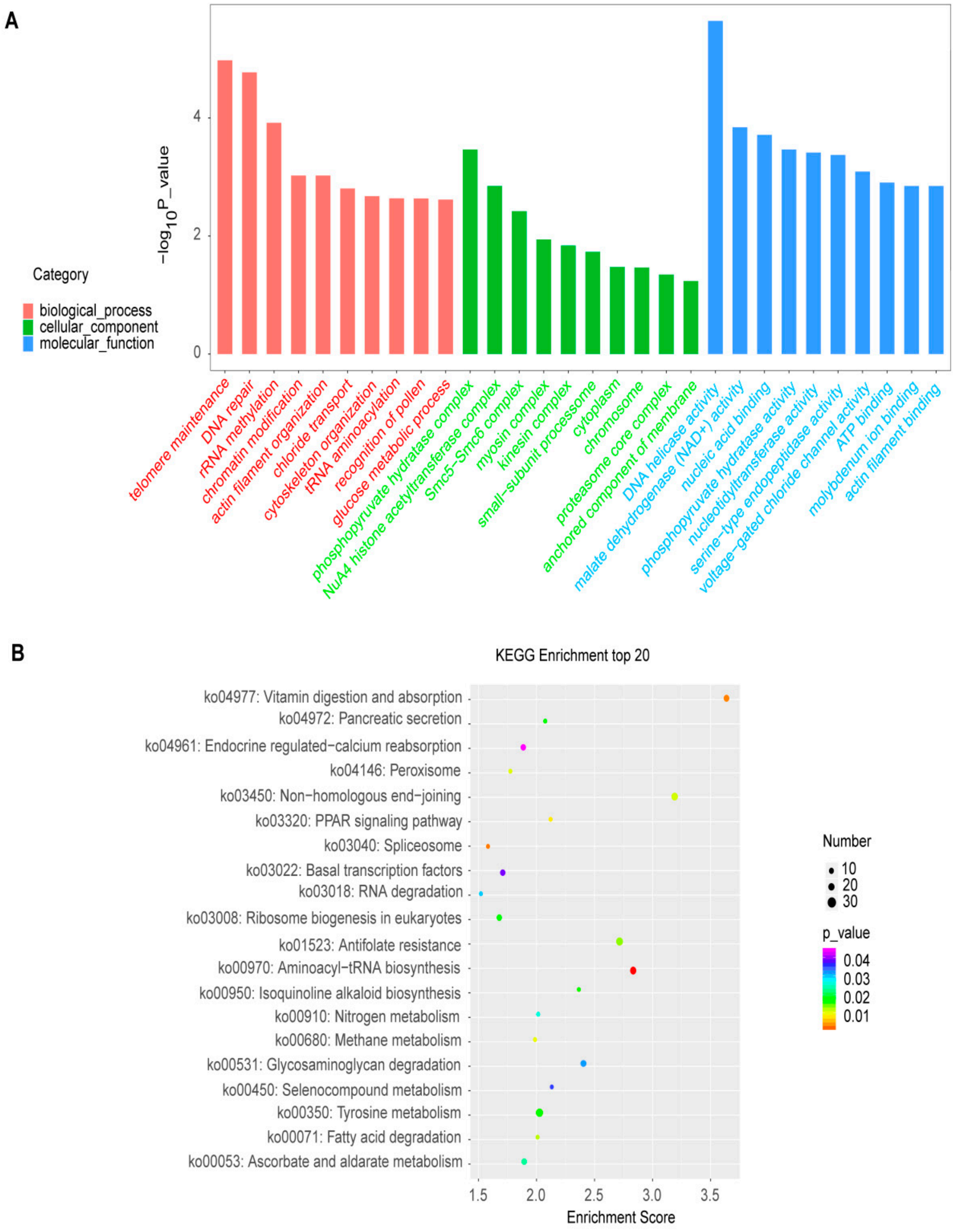

Figure S2: GO and KEGG analyses performed on the ARF14. A, GO analysis displaying the top 20 enriched pathways for the biological process, cellular component and the molecular function. B, KEGG analysis displaying the top 20 enriched metabolic pathways based on the ARF14 gate.

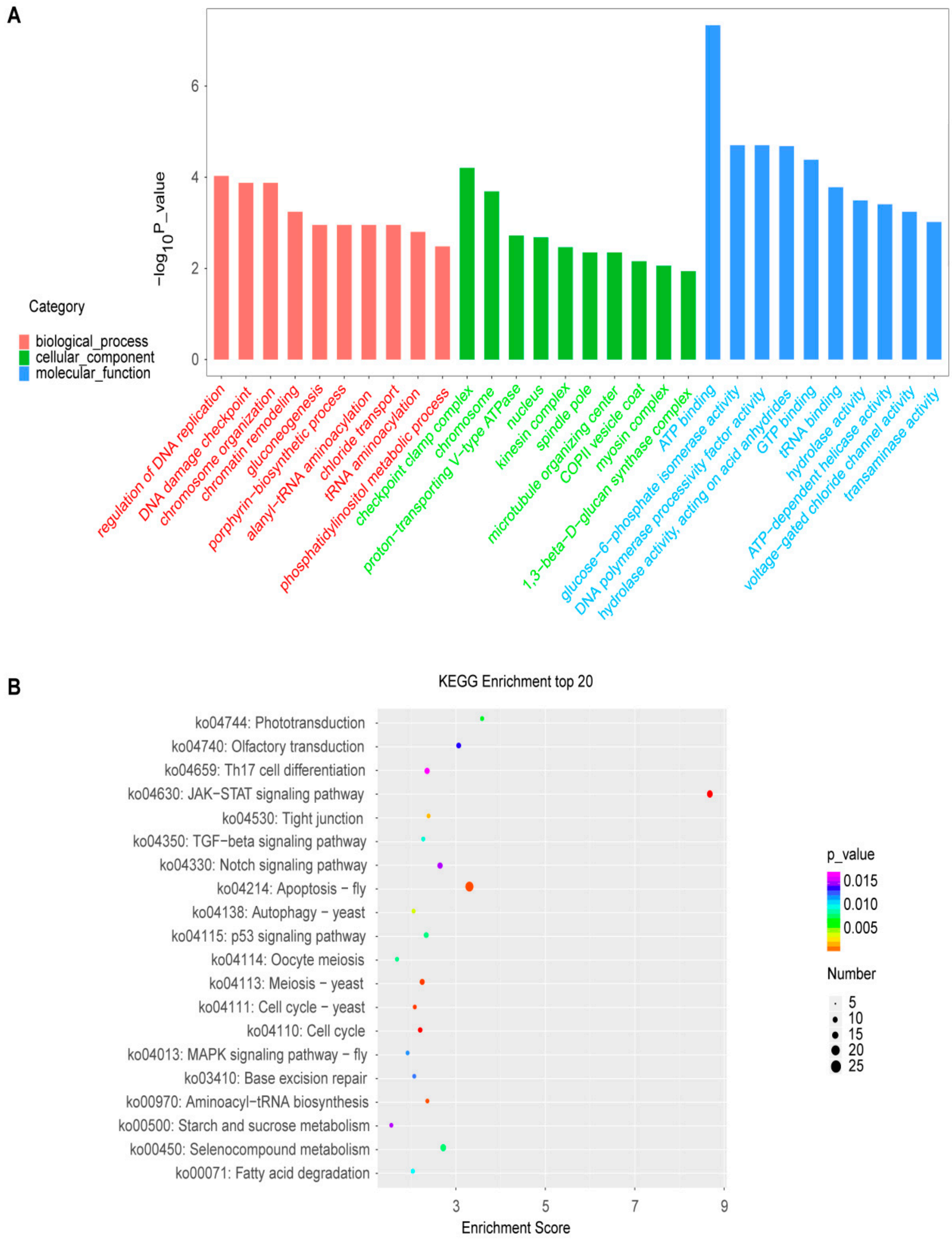

Figure S3: GO and KEGG analyses performed on the ARF18. A, GO analysis displaying the top 20 enriched pathways for the biological process, cellular component and the molecular function. B, KEGG analysis displaying the top 20 enriched metabolic pathways based on the ARF18 gate.

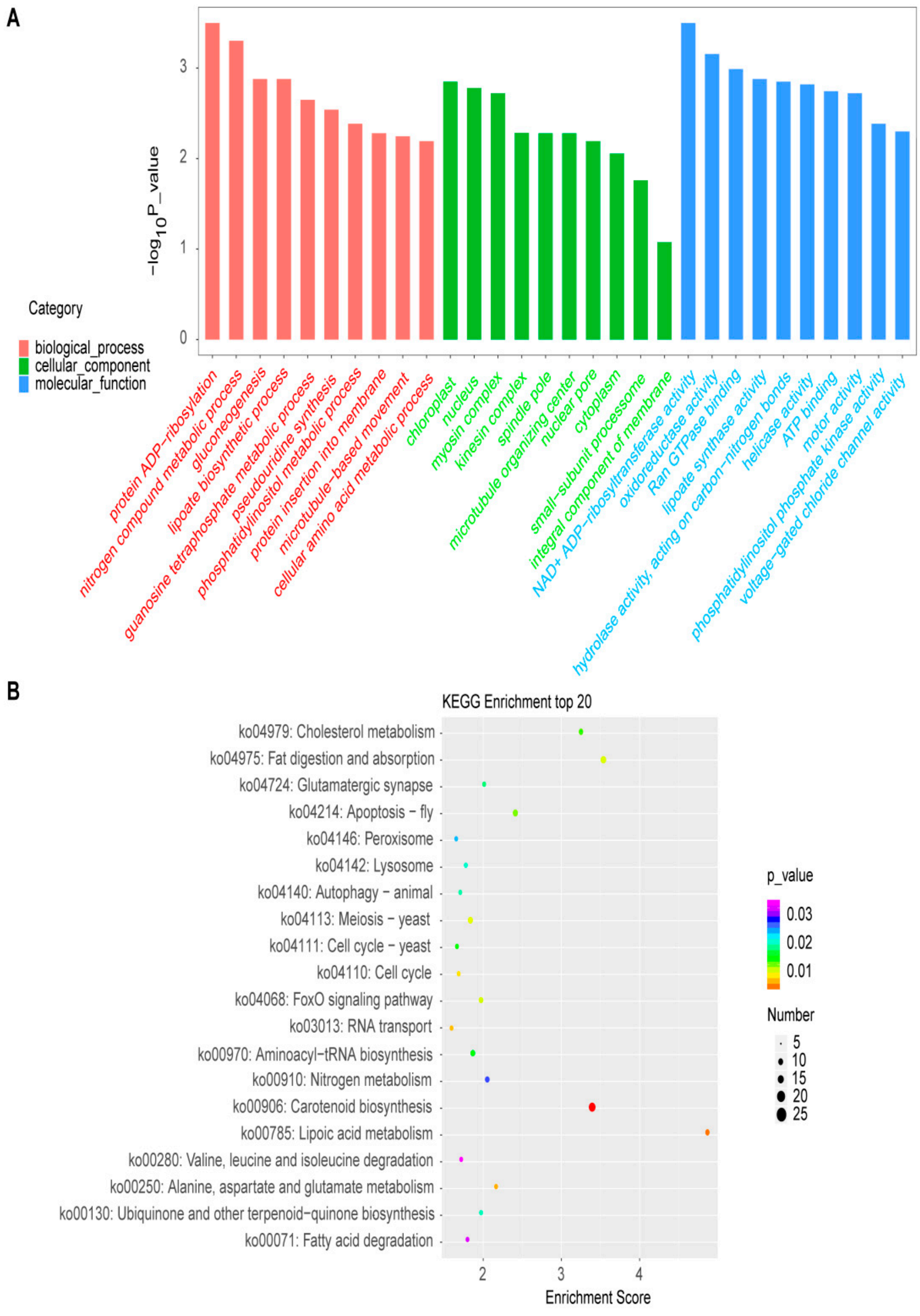

Figure S4: GO and KEGG analyses performed on the ARF35. A, GO analysis displaying the top 20 enriched pathways for the biological process, cellular component and the molecular function. B, KEGG analysis displaying the top 20 enriched metabolic pathways based on the ARF35 gate.
